# Determinants of Improvement in Foot Bimalleolar Angle, Pirani Score, and Recurrence in Clubfoot Patients Treated With the Ponseti Technique

**DOI:** 10.7759/cureus.76344

**Published:** 2024-12-24

**Authors:** Rajesh Kumar, Afroz Ahmed Khan, Abhishek Pandey, Vipin Kumar, Sehaj Singh Kataria

**Affiliations:** 1 Department of Orthopaedics, Institute of Post-Graduate Medical Education and Research and Seth Sukhlal Karnani Memorial Hospital, Kolkata, IND; 2 Department of Orthopaedics, Era's Lucknow Medical College and Hospital, Lucknow, IND; 3 Department of Orthopaedics, Fortis Hospital, Ludhiana, Ludhiana, IND

**Keywords:** clubfoot recurrence, congenital talipes equinovarus, foot bimalleolar angle, pirani score, risk factors of ctev, socioeconomic factors

## Abstract

Introduction: Foot bimalleolar angle (FBM angle) and Pirani score are recognized as assessment and prognostic tools for objective assessment of clubfoot (congenital talipes equinovarus, or CTEV) treatment. The present study proposed to study various factors that determine the improvement in FBM angle and Pirani score in patients treated by the Ponseti technique.

Materials and methods: This prospective observational study was conducted after obtaining ethical clearance, and 42 children with CTEV (60 feet) were enrolled. Various parameters affecting FBM angle and Pirani scoring were recorded for each individual patient on a predesigned proforma. Patients were managed by serial manipulation and casting using the Ponseti technique with weekly follow-ups, and changes in FBM angle and Pirani score were recorded. The result was analyzed, and determinants of improvement in FBM angle, Pirani scoring, and recurrence were compared and reported.

Results: Significant factors affecting FBM angle were age at treatment commencement, severity at presentation, and outside treatment. However, only severity at presentation emerged as the most significant variable. Significant factors affecting Pirani score improvement were age at the start of treatment, severity at presentation, and parent education. However, severity at presentation was the most significant variable. The recurrence of clubfoot was significantly associated with severity at presentation, education of parents, weekly visit compliance, and compliance with orthosis.

Conclusion: The identification of independent risk factors, including various sociodemographic factors affecting CTEV treatment outcomes, can allow clinicians to intervene at various levels of management, including parental counselling and socio-economic support, as measures to enhance compliance and ascertain more promising outcomes.

## Introduction

Hippocrates described congenital talipes equinovarus (CTEV) as one of the most common congenital orthopaedic anomalies in 400 BC [1]. CTEV is detected via routine intrauterine ultrasound of the foetus at around 18-20 weeks of gestation [2]. However, in countries where maternal healthcare services are not institutionalized, it is first diagnosed at delivery [2]. Untreated CTEV persists into adulthood, leading to pain, loss of mobility, stigma, and exclusion from many aspects of daily life, such as education and employment [3]. It has a proclivity to recur, thus continuing to be a challenge. Relapses are reduced after the age of four because the disease that causes CTEV ceases to exist [4]. Treatment is most effective when started early.

CTEV deformity includes foot adduction, supination, and varus positions. Tendons maintain the adduction and inversion of the calcaneus, navicular, and cuboid bones in relation to the talus, which allows them to rotate medially with respect to it. Even though the foot is supinated, the front of the foot is pronated when compared to the back of the foot, resulting in a cavus in the foot. In addition, the first metatarsal is more plantarflexed than the second metatarsal. When occurring in conjunction with other traits as part of a genetic condition, it is referred to as "syndromic." However, it occurs more commonly alone, called "idiopathic CTEV" (ICTEV). ICTEV is associated with joint laxity, congenital dislocation of the hip, tibial torsion, ray anomalies of the foot (oligodactyly), the absence of some tarsal bones, and a history of other foot anomalies in the family [5].

Before commencing treatment, an assessment of the initial degree of deformity is necessary. Treatment of CTEV is typically non-operative in the majority of cases. The International Clubfoot Study Group, established in 2003, has approved Kite's, Ponseti's, and Bensahel's techniques as standardized conservative regimes for the treatment of clubfoot all over the world [6]. Various scoring systems have been proposed to assess the level of deformities [7-10], but lack of objectivity in these systems showed inter-observer variation in findings.

Pirani et al. in 1999 [11] proposed a scoring system that incorporates three components in hindfoot and three in midfoot. This Pirani scoring system is user-friendly, reliable and predictable. It predicts the number of casts required to correct the deformity and the probability of Achilles tendon tenotomy [12,13] and gained popular acceptance. Footprints and podographic foot bimalleolar angle (FBM angle) as suggested by Jain et al. [14] are now being increasingly recognized as an assessment and prognostic tool for objective assessment of clubfoot. The present study was proposed to study the factors which determine the improvement of FBM angle and Pirani score in patients of CTEV treated by the Ponseti technique.

## Materials and methods

In the present prospective observational study, after obtaining institutional ethical clearance and parent’s consent and calculating sample size using statistical techniques with complication rates of 5%, alpha -0.5, and beta of 80%, which came to 60 feet, 42 children who had completed follow-up were assessed between 2019 and 2021. Children treated conservatively elsewhere, including relapse cases and new cases who came for the first time for clubfoot management were included. Previously operated-on cases were excluded from the study. A detailed history, including the determinants of improvement in FBM angle and Pirani scoring and their effect on the recurrence of clubfoot, was recorded for each individual patient on a predesigned proforma and analyzed (Table 1). These sociodemographic details were included in our proforma and methodology after a rigorous discussion and under the guidance of the most senior and experienced faculty members of our institution. Using podograms or plain paper, the infant was placed in a weight-bearing position. FBM angle was calculated at each visit. The Pirani score was calculated at each visit (Table 2). The Ponseti technique of foot correction was applied to all patients. A successful weekly follow-up was done, and any changes in the FBM angle and Pirani score were recorded. This method was adopted to support the effectiveness of the already-proven Poseti technique being used in our institute. Regression analysis was performed to reach a conclusion regarding the effect of various socioeconomic and demographic details on the outcome of treatment using the Ponseti technique. Results were thus analyzed in this manner and determinants of improvement in FBM angle and Pirani scoring in patients with clubfoot treated with the Ponseti technique were compared and reported using multivariate regression analysis. The relationship between these determinants and recurrence in patients with clubfoot was also reported using regression analysis.

**Table 1:** Patient characteristics Table Credits: First Author

Patient characteristics	Frequency	Percentage (%)
Age		
<3 months	35	83.33
>3 months	7	16.67
Gender		
Male	30	71.43
Female	12	28.57
Presenting complaints		
Right clubfoot	14	33.33
Left clubfoot	10	23.81
Bilateral clubfoot	18	42.86
Severity at presentation		
Grade 2	39	65.0
Grade 3	21	35.0
Outside treatment		
Yes	15	37.71
No	27	64.29
Income		
Less than $122 per month	21	50.0
More than $122 per month	21	50.0
Education of parents		
High school or less	18	42.86
More than High school	24	57.14
Siblings		
No	20	47.62
Yes	22	52.38
Plaster break		
No	36	85.71
Yes	6	14.29
Weekly compliance		
No	6	14.29
Yes	36	85.71
Compliance with orthosis		
No	20	47.62
Yes	22	52.38
Recurrence (no. of feet)		
No recurrence	38	63.33
Recurrence	22	36.67

**Table 2:** Calculation of Pirani scoring

	0	0.5	1
Posterior heel crease	Presence of several fine creases	Two or three moderate creases	Single deep crease
Empty heel	Easily palpable calcaneum - not far under the skin	Palpable calcaneum	Calcaneum not palpable
Rigidity of equinus	Dorsiflexion beyond 90°	Foot reaches 90°	Inability of foot to reach 90°
Curvature of lateral border of foot	Foot border straight	Deviation at the level of metatarsal	Deviation at calcaneo-cuboid joint level
Medial crease	Presence of several fine creases	Two or three moderate creases	Single deep crease
Lateral head of talus	Talus completely sinks away under the navicular	Talus moves partially but doesn’t completely sink	Talus moves partially but doesn’t completely sink

Statistical Package for the Social Sciences (IBM SPSS Statistics for Windows, IBM Corp., Version 21, Armonk, NY) was used for the statistical analysis. The number (%) and mean standard deviation were used to present the data. The most significant variable was discovered and presented as a result of using linear regression to look for the univariate linear association first, then the multivariate linear association between a dependent variable and independent variables. The level of significance ("p" is the level of significance) was computed. P-values of less than 0.05 and less than 0.01 were regarded as significant and highly significant, respectively.

## Results

In the current study, out of the total number of patients who arrived at the hospital, 35 (83.33%) of them were before the age of three months, 30 (71.43%) were male, 24 (57.14%) came from families where both parents had a high school diploma, and 22 (52.38%) had siblings. The monthly family income of 21 (50.0%) out of 42 children was less than $122. Only one foot was involved in 24 (57.14%) children, with the right foot alone being involved in 14 (33.3%) and the left foot alone being involved in 10 (23.81%) children. In 18 (42.86%) cases, bilateral involvement was observed. Twenty-seven (64.29%) of the children had not received any previous treatment. The incidence of plaster breaks was observed in only six (14.29%) children. Compliance with orthosis was observed in 22 (52.38%) of children, while compliance with weekly follow-up was observed in 36 (85.71%) (Table 3).

**Table 3:** Foot bimalleolar angle of study population

Time of observation	No. of feet	Min.	Max.	Mean	S.D.
At admission	60	60	79	69.63	3.23
Final assessment	60	80	83	82.22	0.71
Change at final assessment	60	3	14	12.58	3.15
Rate of improvement (per week)	60	0.75	5.50	2.53	0.73

**Figure 1:**
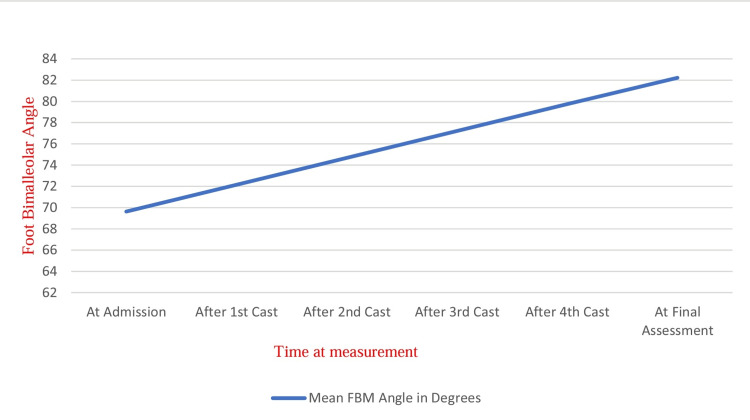
Rate of improvement in foot bimalleolar angle of study population

A total of 60 feet were involved. A recurrence was observed in 22 (36.67%) of the feet. The average FBM angle of children at admission was 69.63±3.23°, but at the final assessment, it was found to be 82.2±0.71°. At the end of the evaluation, the FBM angle had changed by 12.58 ± 3.15°. The rate of weekly improvement in bimalleolar angle was 2.53 ± 0.73° (Table 3 and Figure 1).

**Figure 2:**
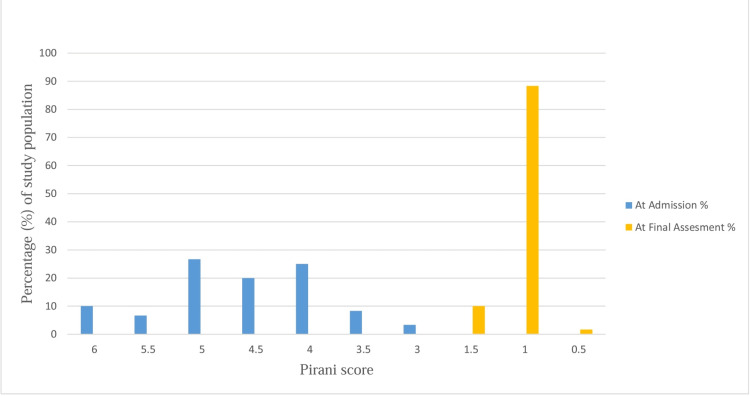
Pirani score by percentage of study population

At admission, the Pirani score of the feet of children ranged from 3.0 to 6.0, with the majority having a Pirani score of ≥4.5 (66.67%). At the final assessment, the Pirani score ranged from 0.5 to 1.5. The majority of the feet had Pirani scores of 1 (88.33%), one (1.67%) foot had a score of 0.5, and the rest, 10.0%, had a score of 1.5 (Figure 2). The median rate of weekly improvement in the Pirani score was 0.7. The mean rate of weekly improvement in the Pirani score was 0.71 ± 0.13.

Multivariate analysis was performed, where FBM angle and Pirani score improvements and recurrence were considered to be dependent on independent variables. Severity at presentation was the only variable that showed a significant association with improvement in FBM angle (Tables 4-5) and Pirani score (Tables 6-7).

**Table 4:** Significance of factors affecting foot bimalleolar angle improvement (univariate analysis)

Variables	‘p’
Gender	0.40
Age at the start of treatment	0.04
Presenting complaint	0.27
Severity at presentation	0.00
Outside treatment	0.05
Income	0.32
Education of parents	0.73
Siblings	0.82
Plaster break	0.31
Weekly visit compliance	0.54
Compliance to orthosis	0.36

**Table 5:** Linear regression of significant variables showing improvement in foot bimalleolar angle (multivariate analysis)

Variable	Coefficient	SE	F-test	‘p’
Severity at presentation	-3.370	1.033	10.635	0.002
Outside treatment	0.679	0.980	0.479	0.493
Start of treatment day	-0.002	0.003	0.306	0.583

**Table 6:** Significance of factors affecting improvement in Pirani score (univariate analysis)

Variables	‘p’
Gender	0.45
Age at the start of treatment	0.04
Presenting complaint	0.10
Severity at presentation	<0.001
Outside treatment	0.10
Income	0.29
Education of parents	0.03
Siblings	0.84
Plaster break	0.93
Weekly visit compliance	0.15
Compliance to orthosis	0.06

**Table 7:** Linear regression of significant variables showing improvement in Pirani score (multivariate analysis)

Variable	Coefficient	SE	F-test	‘p’
Severity at presentation	-0.841	0.235	12.771	0.001
Education of parents	0.198	0.230	0.743	0.394
Age	-0.517	0.342	2.285	0.139
Start of treatment days	0.000	0.001	0.072	0.790
Orthosis compliance	0.170	0.228	0.551	0.462
CONSTANT	-1.719	0.598	8.274	0.007

After performing linear regression on various determinants, the education status of parents, weekly visits, non-compliance with orthosis, and severity at presentation also had a significant effect on the recurrence of clubfoot (Table 8). However, the education of parents turned out to be the most significant factor (Table 9).

**Table 8:** Significance of factors affecting recurrence of clubfoot

Variables	Odds ratio	95% CI	‘p’
Gender	0.81	0.2-3.1	0.38
Age at the start of treatment			0.66
Presenting complaint	-	-	0.85
Severity at presentation	0.25	0.08-0.70 (Protective)	0.01
Outside treatment	0.72	0.19-2.7	0.32
Income	1	0.28-3.47	0.5
Education of parents	18.2	3.7-89.2	<0.001
Siblings	0.81	0.2-3.1	0.38
Plaster break	0.56	0.09-3.2	0.27
Weekly visit compliance	0.06	0.0068-0.060	<0.001
Compliance to orthosis	88	8.9-863	<0.001

**Table 9:** Regression analysis of factors affecting recurrence of clubfoot

Variable	Coefficient	SE	F-test	‘p’
Age of treatment start	-0.175	0.147	1.411	0.242
Education of parents	0.566	0.126	20.262	0.00006
Severity of deformity at presentation	-0.204	0.129	2.497	0.122

## Discussion

CTEV is a profoundly invalidating ailment worldwide; inadequate treatment has drastic consequences for patients' quality of life, with a large social impact. Clubfoot is particularly frequent in developing countries (80% of overall cases). Neglected clubfoot severely restricts the ability to walk in some cases, and in others, only short distances are manageable. Many reports have acknowledged CTEV to be a serious public health issue and that available services are insufficient, resulting in high levels of impairment that may be preventable [15-17].

The current study aimed to investigate the factors that contribute to improvement in FBM angle and Pirani score and their effect on recurrence in patients with CTEV who had undergone treatment with the Ponseti technique. The study included 42 children presenting with CTEV. Ponseti's method of meticulous serial manipulations and cast application is essential to obtaining an initial correction of the idiopathic clubfoot deformity.

As compared to the present study, Singh (2008) [18] included 30 children aged 0-2 years, with 24 (80%) of them being male. In addition, six (40%) of the cases had bilateral involvement. He had reported an increment in FBM angle of 15.5°.

In our study, the age of presentation was mostly less than three months (83.33%). They were mostly males (71.43%). In 57.14% of patients, both feet were affected. A significant improvement of 12.58±3.15° (15.3%) in FBM was observed, comparable to the other study (18).

Jain et al. [19] established the normal FBM angle in Indian infants and correlated it with the severity of deformity and the results of treatment in CTEV. The FBM angle in normal infants was calculated at 82.5 degrees, comparable to the FBM angle achieved at the final assessment in our study.

Anshuman et al. [20] had also treated 24 children using the Ponseti method. The mean Pirani score at initiation was 5.10, which declined to 0.0 at the seven-week follow-up. The increment in FBM angle at the eighth-week follow-up was 14.11°.

The findings of the current study showed an improvement in the Pirani score and FBM angle consistent with the previous studies [19,20]. According to the current study, recurrence of clubfoot was observed in 22 (36.67%) feet and was found to be significantly associated with severity at presentation (odds ratio (OR) = 0.25 and 95% confidence interval (CI): 0.08-0.70; p = 0.01), education of parents (OR = 18.2 and 95% CI: 3.7-89.2; p < 0.001), weekly visit compliance (OR = 0.06 and 95% CI: 0.007-0.06; p < 0.001), and compliance with orthosis (OR = 88 and 95% CI: 8.9-863; p < 0.001). The outcomes of our study have been supported by Azapira et al. [21] and Haft et al. [22].

In this study, we found socio-economic characteristics such as lack of parental education had a negative impact on treatment compliance as well as treatment outcomes for the participants. As a result, it is critical that the problem is not only recognised from a medical standpoint, but also considered from a socio-economic standpoint. According to the researchers, parental counselling and socio-economic support are recommended as approaches to promote compliance to achieve more promising outcomes. Our data showed that noncompliance with the use of the orthosis is the primary risk factor for recurrent deformity.

Our findings showed that the recurrence of clubfoot is not dependent on age at treatment initiation, gender, whether the patient received cast treatment prior to referral, or number of siblings. With the numbers available, the education of parents, severity at presentation compliance to weekly visits, and compliance with orthosis were found to be significant factors. Parental education level in high school or below was found to be a risk factor for relapse. When compared to children whose parents had more than a high school education, these children had a 10-fold increased risk of recurrence. It should be noted that parental income and marital status, which are frequently associated with educational level, were not found to be significant factors in this study; however, both factors were found to be involved in the risk of recurrence. Furthermore, we hypothesize that some of the more educated parents were more motivated to succeed with the Ponseti method and thus more compliant with the use of bracing.

This study’s limitation stems from the fact that it only included patients from middle- and lower-class families in India. We advise multi-centric studies with a larger sample size across numerous nations with various socioeconomic conditions and patient characteristics in order to improve the study’s efficacy. There might also be scope for adding more variables to our list of determinants compiled under the guidance of the most experienced faculty members and further studying their effects. The inaccuracy of the compliance assessment was another drawback of this study. Verbal reports on the use of the brace were the main method of determining compliance. As there was no known objective measurement of compliance, parents may have exaggerated compliance, and some patients may still have received correction despite compliance that was only partial or non-existent.

## Conclusions

Our research adds to the body of knowledge about casting trends and also establishes the significance of socioeconomic and demographic factors influencing FBM angle, Pirani score, and the recurrence of clubfoot. According to the results of our study, each parameter had a different effect on the FBM angle, Pirani score improvement, and the recurrence of clubfoot. Severity at presentation, age at treatment commencement, and outside treatment affected FBM angle improvement. However, severity at presentation emerged as the most significant variable. Improvement in the Pirani score was affected by age at the start of treatment, severity at presentation, and parent education. Out of these determinants, severity at presentation was the most significant. Socioeconomic determinants such as parental education, attendance at weekly visits, use of orthosis, and severity at presentation exhibited a strong correlation in cases of recurrence. Out of these, the education of parents was the most significant. Based on recent research findings, a surgeon can offer resources such as additional education material and home nursing visits to a select group of patients who have a higher risk of treatment failure.
